# *In-Silico* Characterization and *in-Vivo* Validation of Albiziasaponin-A, Iso-Orientin, and Salvadorin Using a Rat Model of Alzheimer's Disease

**DOI:** 10.3389/fphar.2018.00730

**Published:** 2018-08-02

**Authors:** Mahmood Rasool, Arif Malik, Sulayman Waquar, Qura Tul-Ain, Tassadaq H. Jafar, Rabia Rasool, Aasia Kalsoom, Muhammad A. Ghafoor, Sheikh A. Sehgal, Kalamegam Gauthaman, Muhammad I. Naseer, Mohammed H. Al-Qahtani, Peter N. Pushparaj

**Affiliations:** ^1^Center of Excellence in Genomic Medicine Research, Faculty of Applied Medical Sciences, King Abdulaziz University, Jeddah, Saudi Arabia; ^2^Institute of Molecular Biology and Biotechnology, The University of Lahore, Lahore, Pakistan; ^3^Institute of Zoology, Chinese Academy of Sciences, Beijing, China

**Keywords:** Alzheimer's disease, acetylcholinesterase (AChE), salvadorin, Albiziasaponin A, iso-orientin, *in silico* modeling, *in vivo* rat model, molecular docking

## Abstract

Alzheimer's disease (AD) is a neurodegenerative disorder characterized by dementia, excessive acetylcholinesterase (AChE) activity, formation of neurotoxic amyloid plaque, and tau protein aggregation. Based on literature survey, we have shortlisted three important target proteins (AChE, COX2, and MMP8) implicated in the pathogenesis of AD and 20 different phytocompounds for molecular docking experiments with these three target proteins. The 3D-structures of AChE, COX2, and MMP8 were predicted by homology modeling by MODELLER and the threading approach by using ITASSER. Structure evaluations were performed using ERRAT, Verify3D, and Rampage softwares. The results based on molecular docking studies confirmed that there were strong interactions of these phytocompounds with AChE, COX2, and MMP8. The top three compounds namely Albiziasaponin-A, Iso-Orientin, and Salvadorin showed least binding energy and highest binding affinity among all the scrutinized compounds. Post-docking analyses showed the following free energy change for Albiziasaponin-A, Salvadorin, and Iso-Orientin (−9.8 to −15.0 kcal/mol) as compared to FDA approved drugs (donepezil, galantamine, and rivastigmine) for AD (−6.6 to −8.2 Kcal/mol) and interact with similar amino acid residues (Pro-266, Asp-344, Trp-563, Pro-568, Tyr-103, Tyr-155, Trp-317, and Tyr-372) with the target proteins. Furthermore, we have investigated the antioxidant and anticholinesterase activity of these top three phytochemicals namely, Albiziasaponin-A, Iso-Orientin, and Salvadorin in colchicine induced rat model of AD. Sprague Dawley (SD) rat model of AD were developed using bilateral intracerebroventricular (ICV) injection of colchicine (15 μg/rat). After the induction of AD, the rats were subjected to treatment with phytochemicals individually or in combination for 3 weeks. The serum samples were further analyzed for biomarkers such as 8-hydroxydeoxyguanosine (8-OHdG), 4-hydroxynonenal (4-HNE), tumor necrosis factor-alpha (TNF-α), cyclooxygenase-2 (COX-2), matrix metalloproteinase-8 (MMP-8), isoprostanes-2 alpha (isoP-2α), and acetylcholine esterase (AChE) using conventional Enzyme Linked Immunosorbent Assay (ELISA) method. Additionally, the status of lipid peroxidation was estimated calorimetrically by measuring thiobarbituric acid reactive substances (TBARS). Here, we observed a statistically significant reduction (*P* < 0.05) in the oxidative stress and inflammatory markers in the treatment groups receiving mono and combinational therapies using Albiziasaponin-A, Iso-Orientin, and Salvadorin as compared to colchicine alone group. Besides, the ADMET profiles of these phytocompounds were very promising and, hence, these potential neuroprotective agents may further be taken for preclinical studies either as mono or combinational therapy for AD.

## Introduction

Alzheimer's disease (AD) is a progressive and irreversible neurodegenerative disease characterized by dementia and afflicted individuals show a steady decline of memory and cognitive impairment (Zhang et al., 2011). The two pathogenic characteristics of AD are the neuritic plaques (NPs) of β-amyloid protein (Aβ) and insoluble twisted fibers called neurofibrillary tangles (NFTs) in the brain. These neurofibrillary tangles are the aggregates of “Tau” proteins involved in the stabilization of microtubules. Recognizable types of AD are often related with mutations in amyloid precursor proteins (APP) the presenilin-1 (PS1) or presenilin-2 (PS2). Sequential cleavage of APP by γ-secretases leads to the formation of amyloid beta (Aβ) protein, especially their longer isoforms (Aβ_40_, Aβ_42_) and especially Aβ_42_ is more fibrillogenic and is associated with disease states (Yin et al., 2007). β-amyloid protein (Aβ) provokes synaptic disorganization, disturbs neural activity, and induces brain tissue damage. Accumulation and dispersal of Aβ in the brain is often associated with the clinical manifestation of AD (Muliyala and Varghese, 2010). The term AD was initially coined by Emil Kraepelin *in honor of Alois Alzheimer*, a German psychiatrist, who first identified this neurodegenerative disease in 1906 (Möller and Graeber, 1998). Presence of AD may be indicated by co-occurrences such as cognitive dysfunction, hallucinations, anxiety, depression, delusions, irritability, personality changes, sleep disturbance, agitation, restlessness, yelling, shredding paper, poor judgment and difficulty in learning and thoughts (Cummings et al., 1994). Aging Demographics and Memory Study (ADAMS) assessment indicates 16% of females and 11% of males aged 71 or more were suffering with AD (Plassman et al., 2007). The incidence of AD is projected to increase to 135 million by 2050 (He et al., 2016), and an estimate based on the United States 2010 census identified that out of about 5.3 million patients of AD of age group 65, amongst which 3.3 million are women and 2 million are men (Hebert et al., 2013).

AD is a multifactorial neurodegenerative disease due to the accumulation of Aβ plaques and NFTs in the brain. Various genes such as APP, BACE1, PS1/2, ApoE, NEP, IDE are found to be involved in the initiation and development of AD (Dong et al., 2012). Aging is one of the common causative factors for the development of AD. An array of factors are involved in the development and progression of AD like genetic mutation, polymorphism, irregular immune or inflammatory response, injury, oxidative stress, use of drugs, hormone replacement therapies, and also some environmental factors including education, low socio-economic status, nutrition and lack of social interactions (Small, 1998). Lethargy, violence and exertion may exist in these individuals (Förstl and Kurz, 1999). Cognitive dysfunction, diminished memory, difficulty in recognition, impaired speech and gait are predominant features in AD (Sperling et al., 2011). Molecular pathology of disease presents accumulation of amyloid plaques in different areas of brain. Various cutting edge laboratory techniques and tests are essential to understand the associated biological features. The structural and functional brain imaging approaches such as the use of computed tomography (CT), magnetic resonance imaging (MRI), positron emission tomography (PET), and single photon emission computed tomography (SPECT) enables the evaluation of brain activities in general and some also help in identification of pathological lacerations and abrasions in AD (Small, 2002).

Various factors contribute to the efficient treatment of AD and include both pharmacological and non-pharmacological therapies. Currently, there is no definitive therapy for AD. Acetylcholinesterase (AChE) inhibitors are the only licensed drug of various drugs used for the management and treatment of AD, and it helps to recover the symptoms of cognitive and neuropsychiatric impairments in AD. Some non-pharmacological therapies show positive response and attenuate the symptoms of disease (Grossberg et al., 2010). Non-pharmacologic treatments usually preserve and recover cognitive function. They help to maintain behavioral symptoms, personality changes, anxiety, depression, sleep disturbances (Grossberg et al., 2010). Bioactive and naturally occurring phytochemicals are reported to effectively reduce the risks of AD (Essa et al., 2012). Phytochemicals in general, are less toxic as compared to the synthetic drugs (Kim et al., 2014), have many beneficial effects including anti-oxidant activity (Kumar and Khanum, 2012) and therefore can be used for the treatment of AD (Venkatesan et al., 2015). There are some naturally occuring AChE/ butyrylcholinesterase (BChE) inhibitors as well-known as physostigmine and huperzine A from plant origin that show effective cognitive impairment (Essa et al., 2012).

## Materials and methods

### Drugs, chemicals, reagents and assay kits

The Salvadorin and Albiziasaponin A were prepared as described before and the isoorientin was purchased from Sigma (Yoshikawa et al., 2002; Mahmood et al., 2005). All other drugs, chemicals and reagents were purchased from Sigma Chemicals Co. (St. Louis Mo, USA).

### *In Silco* studies

The amino acid sequence of 3 target rat proteins (AChE 614 a.a.), COX2 (614 a.a), and MMP8 (158 a.a) were obtained from Uniprot database in FASTA format with their accession numbers (AChE (1Q83), COX2 (1PXX) respectively. All the proteins were subjected to PSI-BLAST (Altschul et al., 1990) against the Protein Data Bank (Sussman et al., 1998) to recognize the appropriate templates. MODELLER v9.18 (Fiser and Šali, 2003) was utilized to predict the 3D structures of proteins except of MMP8. Structures were further cross-validated with the help of ITASSER (Zhang, 2008). Three-dimensional (3D) structure of MMP8 was retrieved from RCSB (https://www.rcsb.org). Other validation tools used for the validation of protein structures include ERRAT (Colovos and Yeates, 1993), Verify3D (Eisenberg et al., 1997), and Rampage (Lovell et al., 2003). Obtained structures were then minimized using UCSF Chimera 1.112 (Meng et al., 2006) at 1000 steepest and 1000 conjugate gradient runs with Amber force field parameters.

**Figure 1:**
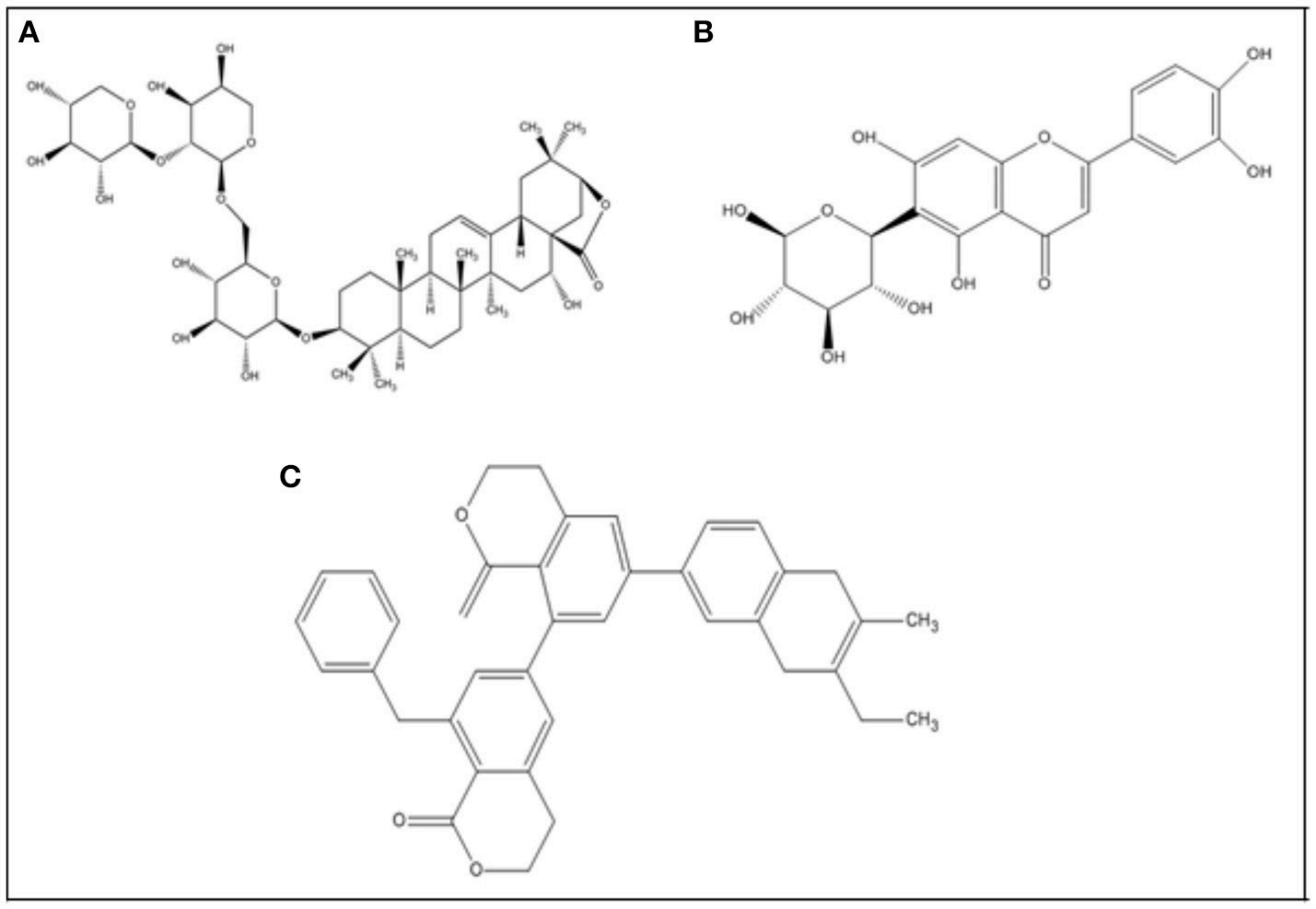
2D chemical structures of selected three top-ranked phytocompounds **(A)**, Albiziasaponin-A **(B)**, Iso-Orientin and **(C)**, Salavadorin.

After extensive survey of literature, 20 phytocompounds were selected from PubChem (Bolton et al., 2008) and were subjected to further structural optimization using ChemDraw Ultra. The energy-minimization, and geometry optimization of all compounds, was carried out by the help of UCSF Chimera v1.12 at 1,500 steepest and 1,500 conjugate gradient runs. The binding sites of all the target proteins were predicted using online tools like COACH (Yang et al., 2013), CASTP (Dundas et al., 2006), and 3D-ligand site (Wass et al., 2010). For comparison, three FDA approved drugs (Donepezil, Galantamine, and Rivastigmine) were administered to rats with AD. Two dimensional (2D) structures of these drugs were retrieved from PubChem (https://pubchem.ncbi.nlm.nih.gov/) and were then configured by ChemDraw ultra (Figure 1). Finally, molecular docking studies were carried out by using Auto Dock Vina (Trott and Olson, 2010). The hydrogen polar atoms were added to all the selected receptor proteins. The total docking runs were sets to 100 for each docking experiment. The grid size was set at 126 × 126 × 126 Å in the x-, y-, and z-axis, respectively, with 0.575 Å grid spacing for all the selected 3 target proteins. The genetic algorithm implemented in Auto Dock Vina was utilized as the key search protocol, while other parameters were set to default values. Further it was visualized by UCSF Chimera v1.12 and ADMET properties of all compounds were calculated by admetSAR online tool (Cheng et al., 2012). The parameters of Lipinski RO5 were calculated by mCule server (Kiss et al., 2012).

### *In Vivo* animal experiments

For the *In vivo* characterization of phytocompounds one hundred (*n* = 100) 6–8 weeks old male Sprague Dawley (SD) rats were categorized into ten different groups (*n* = 10) as A, B, C, D, E, F, G, H, I, and J. Ethical approval from the Institutional Review Board of the University of Lahore was obtained. All the animals were housed in the animal holding unit (AHU) and acclimatized for about 2 weeks under reversed light/dark (12 h each) cycle. Animals were fed on normal rat chow and had access to water *ad libitum*.

### Development of cochicine induced AD model

The SD rats were treated with intracerbroventricualr (icv) injection of cholchicine as described before (Kumar et al., 2009). Briefly, the rats were anasthetized with sodium pentobarbital 45 mg/kg body weight) and placed in stereotaxic apparatus. Through a midline sagittal incison the scalp was reflected and two drill holes made in the skull for placement of the injection canula in the lateral cerebral ventricle. The animals were given post-opeative antibiotic (gentamycin 5 mg/kg, intraperitoneally) to ward off sepsis. Rats were then administered cholchicine (15 μg dissolved in 5 μl of artificial cerebrospinal fluid) using Hamilton microsyringe. To facilitate drug diffusion, the canula was left in place for 2–3 min after the injection. The wound was then sealed with sterile wax and Neosporin powder sprayed externally as an additional antiseptic measure.

### Experimental groups and phytocompounds treatment

The SD rats were randomly divided into 10 different groups (A, B, C, D, E, F, G, H, I, and J) (Table 1). The animals in Group A served as normal control and received no treatment with cholichicine. While the animals in Group B were injected with chochicine, but received no additional treatment and served as positive control. Group C to Group J were treated with the phytocompounds (Albiziasaponin-A, Iso-orientin and Salvadorian) which were selected earlier based on *in silico* screening studies (details provided in the next section). The phytocompounds were administered either individually or in combinations at a concentration of 100 mg/kg for each compound per orally for 3 weeks (see Table 1 for details). Following the study period, all animals were sacrificed using inhalational overdose of carbon dioxide (CO_2_). Blood samples were collected and allowed to clot for 60 min at room temperature. The blood samples were then centrifuged at 3,000 rpm for 10 min and the serum separated were stored as aliquots in −80°C until use in experiments.

**Table 1:** Experimental design.

**Groups (*n* = 10)**	**Treatments**	
	**Cholchicine (15 μg, icv)**	**Phytochemicals (100 mg/kg BW per oral)**
A	No (Sham Control)	Nil
B	Yes (Control)	Nil
C	Yes	Albiziasaponin-A
D	Yes	Iso-Orientin
E	Yes	Salvadorin
F	Yes	Albiziasaponin-A+Iso-Orientin
G	Yes	Albiziasaponin-A+Salvadorin
H	Yes	Iso-Orientin+Salvadorin
I	Yes	Albiziasaponin-A+Salvadorin+Iso-Orientin

### Enzyme linked immunosorbent assay (ELISA)

The serum samples from the control and treatment gorups were analyzed for the levels of 8-hydroxydeoxyguanosine (8-OHdG), 4-hydroxynonenal (4-HNE), tumor necrosis factor-alpha (TNF-α), cyclooxygenase-2 (COX-2), matrix metalloproteinase-8 (MMP-8), isoprostanes-2 alpha (isoP-2α) and acetylcholine esterase (AChE) using commercial ELISA kits according to the respective kit protocol following manufacturer's instructions.

### Lipid peroxidation assay

The level of lipid peroxidation was estimated calorimetrically by measuring thiobarbituric acid reactive substances (TBARS) as described by Ohkawa et al. (1979). Briefly, to 0.2 ml of sample, 8.1% sodium dodecyl sulfate (0.2 ml), 20% acetic acid (1.5 ml) and 0.8% thiobarbituric acid (1.5 ml) were added. Following centrifugation (3,000 rpm for 10 min), the upper organic layer was aspirated, and the optical density (OD) was read at 532 nm using a spectrophotometer (Echelle, LTB Lasertechnik Berlin Gmbh). The levels of lipid peroxides were expressed as millimoles of TBARS/g.

## Statistical analyses

The correlation analysis of the raw data for all the attributes was computed using COSTAT computer package (CoHort software, 2003, Monterey, California). The comparison of means was done by COSTAT computer package using Duncan's Multiple Range (DMR) test.

## Results

### *In Silico* characterization

After the *in-silco* analysis of all the proteins best suitable templates were selected on the basis of identity and query. After the generation of 3D structures of proteins overall identity and query coverage remained >65% in between selected templates and targets from end to end. The percentage was considered satisfactory for the prediction of 3D structure by homology modeling approach. The results were further cross-validated by other approaches using MODELLER V9.18 and ITASSER. Almost about 15 models for each protein were generated and evaluated showing favored, allowed and not-allowed regions. Furthermore, selected models were subjected for molecular docking. With the help of literature survey binding regions of proteins were identified and docked by current literature and various online tools. On basis of the score three best compounds were selected and were then compared with approved drugs for their efficacy.

One hundred runs (100) were done to generate docking complexes out of which top-ranked docked complex was selected for each protein based on the lowest binding affinity. It shows the overall binding energies of selected phytocompounds against AChE, COX2, and MMP8 remain (−6.3 to −15.0 Kcal/mol) as in Table 1. The lowest binding affinity of Albiziasaponin-A against targeted proteins was −13.0, −15.0, and −10.6 Kcal/mol respectively. While in the case of Iso-orientin and Salvadorin, the observed affinities were (−12.5, −11.4 and,−10.0 Kcal/mol) and (−12.5, −12.1, and −9.8 Kcal/mol) respectively. Moreover, these three phytocompounds have the lowest affinities to AChE similar to FDA approved drugs, Donepezil, Galantamine, and Rivastigmine, with binding affinities (−7.8, −8.2, and −6.6 Kcal/mol respectively) as shown in Table 3. All the selected compounds share common interactive residues as listed in Table 4 (Tyr-103, Tyr-155, Trp-317, His-318, Leu320, Glu-323, Phe-328, and Tyr-372). The ADMET profiles (absorption, distribution, metabolism, excretion, and toxicity) also differed significantly as given in Table 5. The comparative molecular docking analyses of top 3 selected compounds and FDA approved drugs against AChE and the potential binding modes of these compounds with the interacting aminoacid residues at the atomic level with AChE were give in Tables 6, 7 respectively. Besides, we have depicted the specific atoms of these three phytocompounds interacting with the aminoacid residues in the binding site of AChE in Figure 5.

**Table 2:** Response of albiziasaponin-a, salvadorin, iso-orientin in a rat model following colchicine (col) induced oxidative stress.

**Groups**	**Mean** ± **SD (*****n*** = **10)**							
	**AChE μmol/min/mg Protein**	**4-HNE (ng/L)**	**8-OHdG (pg/ml)**	**TNF-α (ng/ml)**	**IsoP-2α (pg/ml)**	**MDA (nmol/ml)**	**COX-2 (ng/ml)**	**MMP-8 (ng/ml)**
A	1.93 ± 0.03	1.29 ± 0.016	2.09 ± 0.16	18.29 ± 1.88	21.25 ± 2.19	0.99 ± 0.056	0.71 ± 0.012	33.25 ± 2.08
B	3.19 ± 0.95	18.26 ± 1.29	21.29 ± 3.29	92.26 ± 3.28	181.26 ± 5.26	8.28 ± 1.26	4.29 ± 1.07	115.26 ± 12.26
C	2.09 ± 0.62	12.29 ± 2.22	17.19 ± 2.28	45.29 ± 4.56	102.26 ± 7.28	4.29 ± 2.16	2.08 ± 0.99	65.26 ± 5.16
D	2.03 ± 0.19	16.19 ± 3.19	15.29 ± 3.29	56.23 ± 4.09	98.26 ± 6.25	5.99 ± 1.09	3.09 ± 0.19	71.26 ± 12.25
E	1.99 ± 0.23	13.29 ± 2.55	13.29 ± 2.18	32.26 ± 4.26	78.26 ± 7.26	6.66 ± 2.88	1.99 ± 0.166	52.26 ± 3.88
F	1.86 ± 0.13	10.26 ± 4.26	8.89 ± 1.28	35.26 ± 4.26	47.26 ± 5.26	4.19 ± 1.08	2.58 ± 0.19	46.26 ± 4.26
G	1.86 ± 0.11	12.26 ± 4.16	14.26 ± 3.29	27.19 ± 3.29	68.26 ± 4.44	3.29 ± 1.07	2.99 ± 0.198	51.26 ± 6.35
H	1.74 ± 0.18	9.28 ± 2.11	8.29 ± 3.26	28.26 ± 5.26	45.29 ± 4.23	4.19 ± 1.00	1.99 ± 0.165	50.26 ± 6.25
I	1.66 ± 0.32	2.16 ± 1.08	3.29 ± 1.99	15.26 ± 3.26	27.26 ± 4.277	1.09 ± 0.087	1.06 ± 0.047	40.22 ± 6.32
LSD (0.05)	0.34	3.02	6.29	10.26	8.16	2.09	1.25	7.16
*p*-VALUE	0.034	0.001	0.014	0.013	0.012	0.030	0.000	0.019

*A-Control; B-Col alone; C-Col+Albiziasaponin-A; D-Col+Iso-Orientin; E-Col+Salvadorin; F-Col+Albiziasaponin-A+Iso-Orientin. G-Col+Albiziasaponin-A+Salvadorin; H-Col+Iso-Orientin+Salvadorin; I-Col+Albiziasaponin-A+Salvadorin+Iso-Orientin. Dose of Colchicine (Col) (15 μg Intracerebroventricular injection in each animal). Dose of Albiziasaponin-A, Salvadorin, Iso-Orientin (100 mg/kg BW per oral)*.

**Table 3:** Pearson s' correlation coeeficients of different variables in rats under colchicine (col) stress receiving albiziasaponin-a, salvadorin, iso-orientin.

**Variables**	**AchE**	**4-HNE**	**8-OHdG**	**TNF-α**	**IsoP-2α**	**MDA**	**COX-2**	**MMP-8**
AchE	1.000	0.423	0.519	0.399	0.435	0.512*	0.423	0.823**
4-HNE		1.000	0.645*	0.715**	0.619**	0.774**	0.684**	0.659*
8-OhdG			1.000	0.648**	0.671**	0.719**	0.589*	0.726**
TNF-α				1.000	0.746*	0.659**	0.589**	0.865**
IsoP-2α					1.000	0.614**	0.741**	0.665
MDA						1.000	0.621*	0.596**
COX-2							1.000	0.619*
MMP-8								1.000

**Correlation is significant at the 0.01 level (two-tailed). *p < 0.05, **p < 0.01, ***p < 0.001.

**Table 4:** Binding affinities of all 20 phytocompounds and mCule properties.

**Phytocompounds**	**Binding affinities (kcal/mol) against ache**	**Binding affinities (kcal/mol) against cox2**	**Binding affinities (kcal/mol) against MMP8**	**Mass**	**LOGP**	**HBA**	**HBD**	**PSA**	**RO5 Violations**	**Atoms**	**Rings**
Salvadorin	−12.5	−12.1	−9.8	414.62	6.34	3.00	0	43.37	1	72.00	5
Albiziasaponin-A	−13.0	−15.0	−10.6	897.05	0.40	17	9	263.75	3	135	9
Epigallocatechin-3-gallate	−11.5	−10.6	−10.6	458.37	2.23	11	8	197.37	2	51	4
β-Sitosterol	−11.9	−8.7	−8.3	432.76	8.74	1	1	20.23	1	87	4
Iso-orientin	−12.5	−11.4	−10.0	434.34	−0.24	11	8	201.28	2	49	4
Melanoxetin	−9.9	−10.3	−7.9	302.23	1.98	7	5	131.36	0	32	3
Epicatechin	−9.2	−8.9	−7.8	290.26	1.54	6	5	110.38	0	35	3
Albigenin	−10.5	−9.8	−7.9	426.67	7.10	2	1	37.300	1	77	5
Lupeol	−9.6	−9.1	−7.3	426.71	8.02	1	1	20.23	1	81	5
Catechin	−10.0	−9.5	−8.5	290.26	1.54	6	5	110.38	0	35	3
Cabralealactone	−9.4	−10.1	−7.1	552.70	8.69	3.00	1.00	35.30	2.00	78.00	7.00
β-Amyrin	−8.9	−10.8	−7.5	426.71	8.16	1	1	20.23	1	81	5
Isovitexin	−9.9	−9.1	−9.3	132.37	0.09	10	7	181.05	1	51	4
Oleanolic acid	−9.4	−10.8	−8.2	456.69	7.23	3	2	57.53	1	81	5
Elliptone	−10.0	−9.1	−8.7	352.33	3.56	6	0	67.13	0	42	5
Genistein	−7.6	−9.4	−6.3	270.23	2.57	5	3	90.90	0	30	3
Kaempferol	−9.0	−8.1	−6.6	286.23	2.28	6	4	111.13	0	31	3
Solasodine	−8.1	−10.1	−7.1	429.68	5.57	3.00	1.00	32.70	1.00	78.00	6.00
Afzelechin	−7.1	−7.9	−7.5	274.26	1.84	5	4	90.15	0	34	3
Luteolin	−9.4	−10.3	−7.2	286.23	2.28	6	4	111.13	0	31	3

**Table 5:** ADMET profile analyses of all 20 phytocompounds.

**Phytocompounds**	**Bbb**	**Hia**	**CYP450 2C9 and 2D6 substrate**	**CYP450 2C9 and 2D6 inhibitor**	**CYP Inhibitory promiscuity (IP)**	**Ames toxicity**	**Carcinogens**	**Biodegradation**	**Acute oral toxicity**	**Aqueous solubility (LogS)**	**Rat acute toxicity (LD50, mol/kg)**
**Salvadorin**	+	+	Non-substrate	Non-inhibitor	Low CYP IP	Non AMES toxic	Non- Carcinogens	Not readily biodegradable	III	−5.3955	2.0387
Albiziasaponin-A	+	+	Non-substrate	Non-inhibitor	Low CYP IP	Non AMES toxic	Non- Carcinogens	Not readily biodegradable	III	−4.2181	3.5521
Epigallocatechin-3-gallate	–	+	Non-substrate	Non-inhibitor	Low CYP IP	Non-AMES Toxic	Non- Carcinogens	Not readily biodegradable	IV	−3.3141	2.6643
β-Sitosterol	+	+	Non-substrate	Non-inhibitor	Low CYP IP	Non AMES toxic	Non- Carcinogens	Not readily biodegradable	I	−4.7027	2.6561
Iso-orientin	–	+	Non-substrate	Non-inhibitor	Low CYP IP	AMES Toxic	Non- Carcinogens	Not readily biodegradable	IV	−2.3978	2.3664
Melanoxetin	+	+	Non-substrate	Inhibitor for CYP450 2C9 and Non-inhibitor for CYP450 2D6	Low CYP IP	Non-AMES Toxic	Non- Carcinogens	Not readily biodegradable	II	−3.0804	3.1831
Epicatechin	–	+	Non-substrate	Non-inhibitor	Low CYP IP	Non-AMES Toxic	Non- Carcinogens	Not readily biodegradable	IV	−3.1015	1.8700
Albigenin	+	+	Non-substrate	Non-inhibitor	Low CYP IP	Non AMES toxic	Non- Carcinogens	Not readily biodegradable	III	−4.0877	2.0616
Lupeol	+	+	Non-substrate	Non-inhibitor	Low CYP IP	Non AMES toxic	Non- Carcinogens	Not readily biodegradable	III	−4.4139	3.3838
Catechin	–	+	Non-substrate	Non-inhibitor	Low CYP IP	Non-AMES Toxic	Non- Carcinogens	Not readily biodegradable	IV	−3.1015	1.8700
Cabralealactone	+	+	Non-substrate	Non-inhibitor	Low CYP IP	Non AMES toxic	Non- Carcinogens	Not readily biodegradable	III	−4.0522	2.4518
β-Amyrin	+	+	Non-substrate	Non-inhibitor	Low CYP IP	Non AMES toxic	Non- Carcinogens	Not readily biodegradable	III	−4.5209	2.0842
Isovitexin	–	+	Non-substrate	Non-inhibitor	Low CYP IP	AMES Toxic	Non- Carcinogens	Not readily biodegradable	IV	−2.3978	2.3664
Oleanolic acid	+	+	Non-substrate	Non-inhibitor	Low CYP IP	Non AMES toxic	Non- Carcinogens	Not readily biodegradable	III	−4.3883	2.3902
Elliptone	+	+	Non-substrate	Inhibitor	High CYP IP	Non AMES toxic	Non- Carcinogens	Not readily biodegradable	III	−3.2813	2.4560
Genistein	+	+	Non-substrate	Inhibitor for CYP450 2C9 and Non-inhibitor for CYP450 2D6	High CYP IP	Non AMES toxic	Non- Carcinogens	Not readily biodegradable	II	−3.0925	3.2988
Kaempferol	+	+	Non-substrate	Inhibitor for CYP450 2C9 and Non-inhibitor for CYP450 2D6	High CYP IP	Non AMES toxic	Non- Carcinogens	Not readily biodegradable	II	−3.1423	3.0825
Solasodine	+	+	Non-substrate	Non-inhibitor	Low CYP IP	Non AMES toxic	Non- Carcinogens	Not readily biodegradable	III	−4.0047	1.9513
Afzelechin	+	+	Non-substrate	Non-inhibitor	Low CYP IP	Non AMES toxic	Non- Carcinogens	Not readily biodegradable	IV	−3.2332	2.0532
Luteolin	–	+	Non-substrate	Non-inhibitor	High CYP IP	Non AMES toxic	Non- Carcinogens	Not readily biodegradable	II	−2.9994	3.0200

**Table 6:** Comparative molecular docking analyses of top 3 selected compounds and FDA approved drugs against AChE.

**TOP 3 selected phytocompounds from 20 phytocompounds and FDA approved drugs**	**BINDING affinities (kcal/mol) of top 3 selected phytocompounds and FDA approved drugs**	**INTERACTIVE residues in docked complexes of top 3 phytocompounds and FDA approved drugs**
Albiziasaponin-A	−13.0 Kcal/mol	Asn-264, Pro-266, Thr-269, Ser-271, Arg-327, Thr-342, Asp-344, Trp-563, Asn-564, Pro-568, Leu-571
Iso-orientin	−12.5 Kcal/mol	**Tyr-103, Tyr-155, Trp-317, His-318, Leu320, Glu-323, Phe-328**, Tyr-368, Phe-369, **Tyr-372**
Salvadorin	−12.5 Kcal/mol	**Tyr-103, Tyr-155, Trp-317, His-318, Leu320, Glu-323, Phe-328, Tyr-372**
**FDA APPROVED DRUGS**		
Donepezil	−7.8 Kcal/mol	Pro-266, Asp-344, Gln-444, Pro-441, His-436, Trp-563, Pro-568
Galantamine	−8.2 Kcal/mol	Tyr-103, Tyr-155, Trp-317, Ser-324, Tyr-372
Rivastigmine	−6.6 Kcal/mol	Gly-45, Pro-83, Leu-209, Gln-212, Trp-213, Glu-216

**Table 7:** The binding modes of these compounds with the interacting aminoacid residues at the atomic level with AChE.

**Top 3 selected phytocompounds**	**Compounds interact with atoms of the active site residues of AChE**
Albiziasaponin-	Asn-264: CA, CB, CG, OD1, HD21, ND2 Pro-266: 1C, CA, CG, N Thr-269: CA, CB, CG2, HG1, OG1 Ser-271: CA, CB, HG, OG Arg-327: CA, CB, CG, CZ, HE, HH11, HH12, H21, H22, NH1, NH2, NE Thr-342: CA, CB, HB, H1,H21, H22, H23, OG1, Asp-344: CA, CB, HB2, HB3, OD1, OD2 Trp-563: CA, CB, CD1, CD2, CE2, CE3, CH2, CG, CZ2, CZ3, HE1, NE1 Asn-564: CA, CB, CG, HB2, HB3, HD22, OD1, OE1, ND2 Pro-568: CB, CD, CG, HB2, HB3, HD2, HD3, HG2, HG3, N Leu-571: CA, CB, CG, CD1, CD2, HG, HB2, HB3, HD12, HD13, HD22, HD23
Iso-orientin	Tyr-103: CA, CB, CG, CD2, CE1, CE2, CZ, HH, OH Trp-317: CA, CB, CG, CD1, CD2, CE1, CE2, CE3, CH2, CZ2, CZ3, HB2, HB3, HD1, HE1, HE3, HH2, HZ2, HZ3 Tyr-155: CA, CB, CG, CD1, CD2, CE1, CE2, CZ, HH, OH His-318: CA, CB, CG, CD2, CE1, HB3, HE1, HE2, HD2, ND1, NE2 Leu320: CA, CB, CG, CD1, CD2, HG, HB2, HB3, HD11, HD12, HD13, HD21, HD22, HD23 Glu-323: CB, CG, CD, HB2, HB3, HG2, HG3, OE1, OE2 Phe-328: CA, CB, CG, CD1, CD2,CE1, CE2, CZ, HB2, HB3, HD1, HD2, HE1, HE2 Tyr-368: CA, CB, CG, CD1, CD2, CE1, CE2, CZ, HH, OH Phe-369: CB, CG, CA, CD1, CD2, CE1, CE2, CZ, HB2, HB3, HD1, HD2, HE1, HE2, Tyr-372: CB, CG, CD1, CD2, CE1, CE2, CZ, HB2, HB3, HD1, HD2, HE1, HE2, HH, OH
Salvadorin	Tyr-103: CA, CB, CG, CD1, CD2, CE1, CE2, CZ, HB2, HB3, HD1, HD2, HH, OH Tyr-155: CA, CB, CG, CD1, CD2, CE1, CE2, CZ, HH, OH Trp-317: CA, CB, CG, C, CD1, CD2, CE1, CE2, CE3, CH2, CZ2, CZ3, HB2, HB3, HD1, HE1, HE3, HH2, HZ2, HZ3, NE1 His-318: CA, CB, CG, CD2, CE1, HB2, HB3, HE1, HE2, HD2, ND1, NE2 Leu320: CA, CB, CG, CD1, CD2, HG, HB2, HB3, HD11, HD12, HD13, HD21, HD22, HD23 Glu-323: CA, CB, CG, CD, HB2, HB3, HG2, HG3, OE1, OE2 Phe-328: CA, CB, CG, CD1, CD2,CE1, CE2, CZ, HB2, HB3, HD1, HD2, HE1, HE2 Tyr-372: CA, CB, CG, CD1, CD2, CE1, CE2, CZ, HB2, HB3, HD1, HD2, HE1, HE2, HH, OH

## *In Vivo* studies

The current study showed that use of phytocompounds individually or in combination have exerted significant improvements in biochemical parameters in the rat model of AD. Colchicine (Col) is responsible for the induction oxidative stress (Table 2) when compared to rats receiving colchicine presented the levels of AChE, 4-HNE, 8-OHdG, TNF-α, Iso-P2α, MDA, COX-2 and MMP-8 were significantly higher (3.19 ± 0.95 μmol/min/mg protein, 18.26 ± 1.29 ng/L, 21.29 ± 3.29 pg/ml, 92.26 ± 3.28 ng/ml, 181.26 ± 5.26 pg/ml, 8.28 ± 1.26 nmol/ml, 8.28 ± 1.26 nmol/ml, 4.29 ± 1.07 ng/ml, and 115.26 ± 12.26 ng/ml) as compared to the control group (1.93 ± 0.03 μmol/min/mg protein, 1.29 ± 0.016 ng/L, 2.09 ± 0.16 pg/ml, 18.29 ± 1.88 ng/ml, 21.25 ± 2.19 pg/ml, 0.99 ± 0.056 nmol/ml, 0.71 ± 0.01 ng/ml, and 33.25 ± 2.08 ng/ml). Furthermore, it shows that rats receiving Albiziasaponin-A, Iso-orientin and Salvadorian individually in Group C, D, and E reduced the levels of oxidative stress markers. Levels of 4-HNE and MDA were maximally reduced in the group C (receiving Albizasaponin-A) with (12.29 ± 2.22 ng/L) and (4.29 ± 2.16 nmol/ml) followed by group D and E (16.19 ± 3.19 ng/L, 5.99 ± 1.09 nmol/ml) and (13.29 ± 2.55 ng/L, 6.66 ± 2.88 nmol/ml) respectively. While levels of 8-OHdG, TNF-α and IsoP-2α were most improved in group E. Groups F, G and H were given different combinations of these phytocompounds. Results show a maximum synergism in the group H (group treated with combination of Iso-Orientin and Salvadorian, and results were significant as compared to all other groups (B, C, D, E, F, G). Finally in group I (treated with all three phytochemicals Albiziasaponin-A, Iso-orientin and Salvadorian) levels of different biochemical markers (4-HNE, 8-OHdG, TNF-α, IsoP-2α and MDA) were significantly reduced (2.16 ± 1.08 ng/L, 3.29 ± 1.99 pg/ml, 15.26 ± 3.26 ng/ml, 27.26 ± 4.277 pg/ml, and 1.09 ± 0.087 nmol/ml) as compared to group B (colchicine alone) and all the treatment groups C, D, E, F, G, and H. A significant positive correlation was observed among different variables, AChE vs. MMP-8 (*r* = 0.823^**^), TNF-α vs. MMP-8 (*r* = 0.865^**^), 8-OHdG vs. MDA (*r* = 0.719^**^), and 4-HNE vs. MDA (*r* = 0.774^**^) in rats experimentally induced with colchicine and administered with Albiziasaponin-A, Iso-Orientin and Salvadorin (Table 3).

## Discussion

The field of drug designing and development has progressed over last few years. It elucidates new and useful computational methods for the development of novel drugs (Kumar et al., 2011). In silico studies enabled the researchers to identify and develop less toxic herbal medicines as compared to that of conventional remedies (Taylor et al., 2001). The present study was designed to characterize the beneficial effects of different phytocompounds against AD using both *in silico* and *in vivo* strategies. Several phytocompounds with different active groups were screened and characterized using molecular docking studies. The top three phytocompounds, Albiziasaponin-A, Iso-Orientin, and Salvadorin, were selected for further validation in a rat model of AD based on least binding energy and highest binding affinity with target proteins, AChE, COX2, and MMP8, as compared to other phytocompounds. Moreover, Albiziasaponin-A, Iso-Orientin, and Salvadorin interact with the amino acid residues in the binding sites of AChE similar to the FDA approved drugs (donepezil, galantamine and rivastigmine) for AD treatment. Also, the cross validation of binding sites of the selected target proteins using literature mining precisely envisage the binding sites were similar to the binding pocket identified in our molecular docking analyses (Cheung et al., 2012, 2013; Caliandro et al., 2018). Besides, other phytocompunds, such as Epigallocatechin-3-Gallate (EGCG), and β-Sitosterol, strongly bind *in silico* with AChE, COX2, and MMP8. The EGCG has a very strong antioxidant activity, which is ascribed to the presence of B ring trihydroxy group and esterified gallate in C3 of the ring and it may cross the blood-brain barrier (BBB) in a time-dependent manner (Kim et al., 2014). The EGCG binds with proteins in the plasma membrane and modulates signal transduction pathways, expression of transcription factors, DNA methylation, mitochondrial function, and autophagy to cause its biological actions (Alam and Khan, 2014; Kim et al., 2014; Sehgal et al., 2016; Jamil et al., 2017; Yousuf et al., 2017). The signaling pathways regulated by EGCG include protein kinase C (PKC), NF-kB, and mitogen-activated protein kinase (MAPK) pathway (Kwon et al., 2012; Kim et al., 2014). The EGCG attenuates the activation of NF-kB, c-jun N-terminal kinase and MAPK p38 phosphorylation (Venkatesan et al., 2015). It was shown that the reduction in the release of nitric oxide (NO) by EGCG supresses the MAPK pathways in neuroblastoma cells leading to substantial decrease in both inflammation and oxidative stress levels (Kennedy et al., 2014)

Recent studies demonstrate the effects of phenolic compounds on APP in cell cultures through the inhibition of AChE and BChE to attenuate the formation of β amyloid plaques (Ahmad et al., 2017; Ayaz et al., 2017a,b). It was reported that β-sitosterol inhibits AChE activity both *in vivo* and *in vitro* (Ayaz et al., 2017a,b). It was further deduced that β-sitosterol can easily cross blood brain barrier and moves to the part of brain involved in cognition and inhibit the degradation of acetyl choline (Ach) mediated by AChE (Ayaz et al., 2017a,b). Hence, the inhibition of AChE and BChE may be considered as the primary reason for the degradation of essential neurotransmitter (ACh) (Ali et al., 2017). Therefore, the development of drugs that inhibit AChE and BChE may serve as one of the most useful options to attenuate the progression of AD.

**Figure 2:**
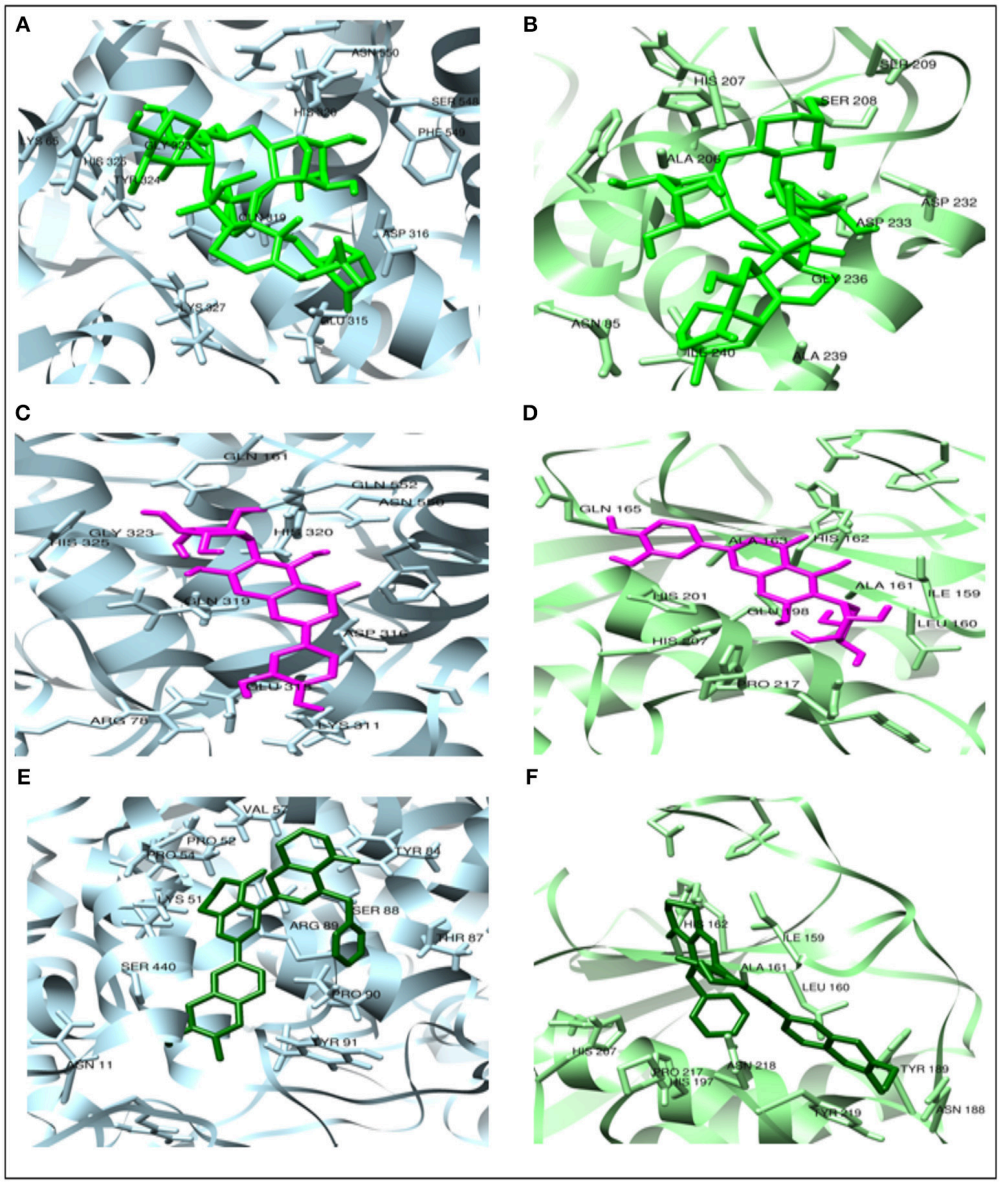
The docked complexes of COX2 (light blue) and MMP2 (light green). Top-ranked 3 phytocompounds **(A,B)** Albiziasaponin-A (green), **(C,D)** Iso-orientin (magenta), and **(E,F)** Salvadorin (dark green).

**Figure 3:**
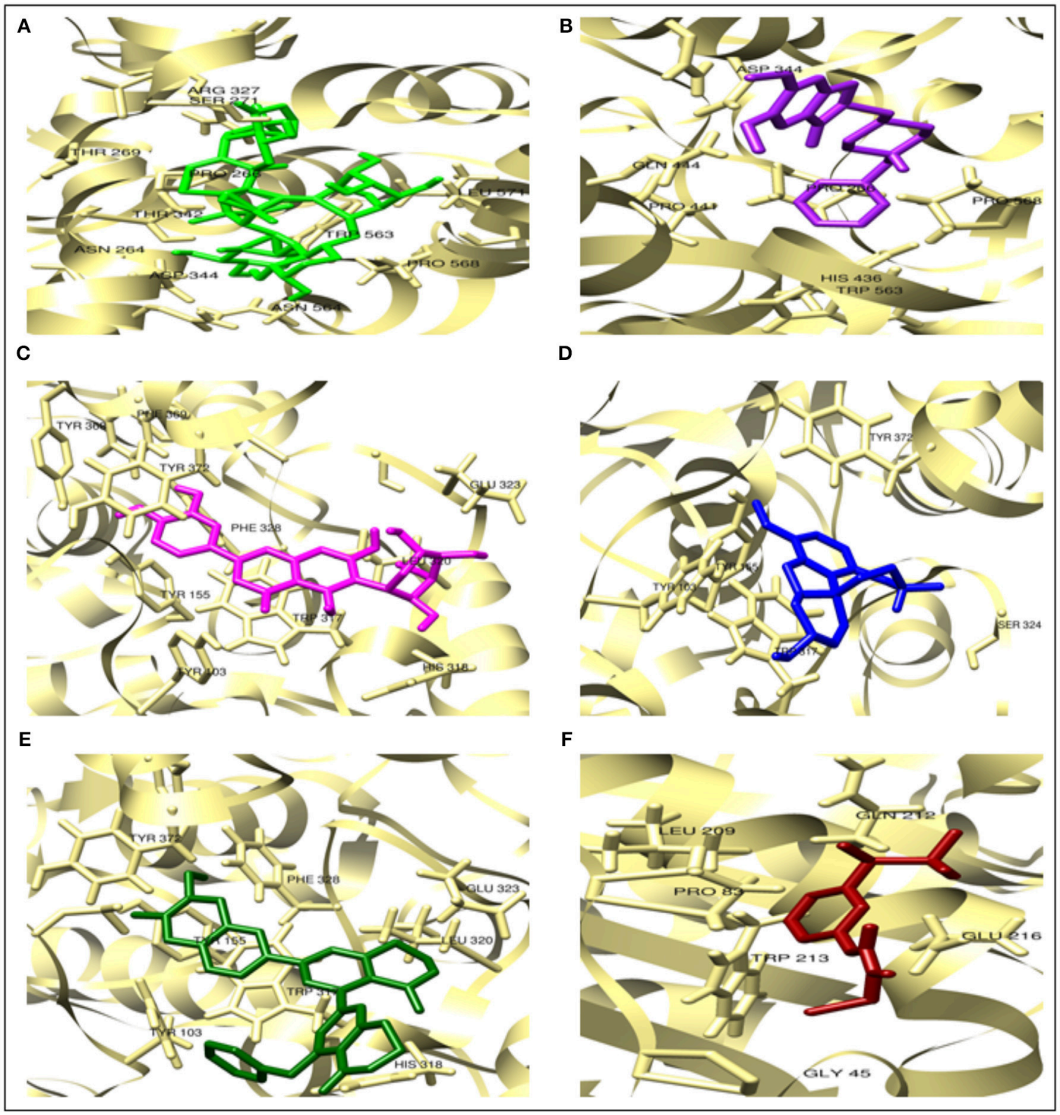
The comparative docked complexes of AChE (khaki). Top-ranked 3 phytocompounds **(A)** Albiziasaponin-A (green), **(C)** Iso-orientin (magenta), **(E)** Salvadorin (dark green) and FDA approved drugs **(B)** donepezil (purple), **(D)** galantamine (blue) and **(F)** rivastigmine (dark red).

**Figure 4:**
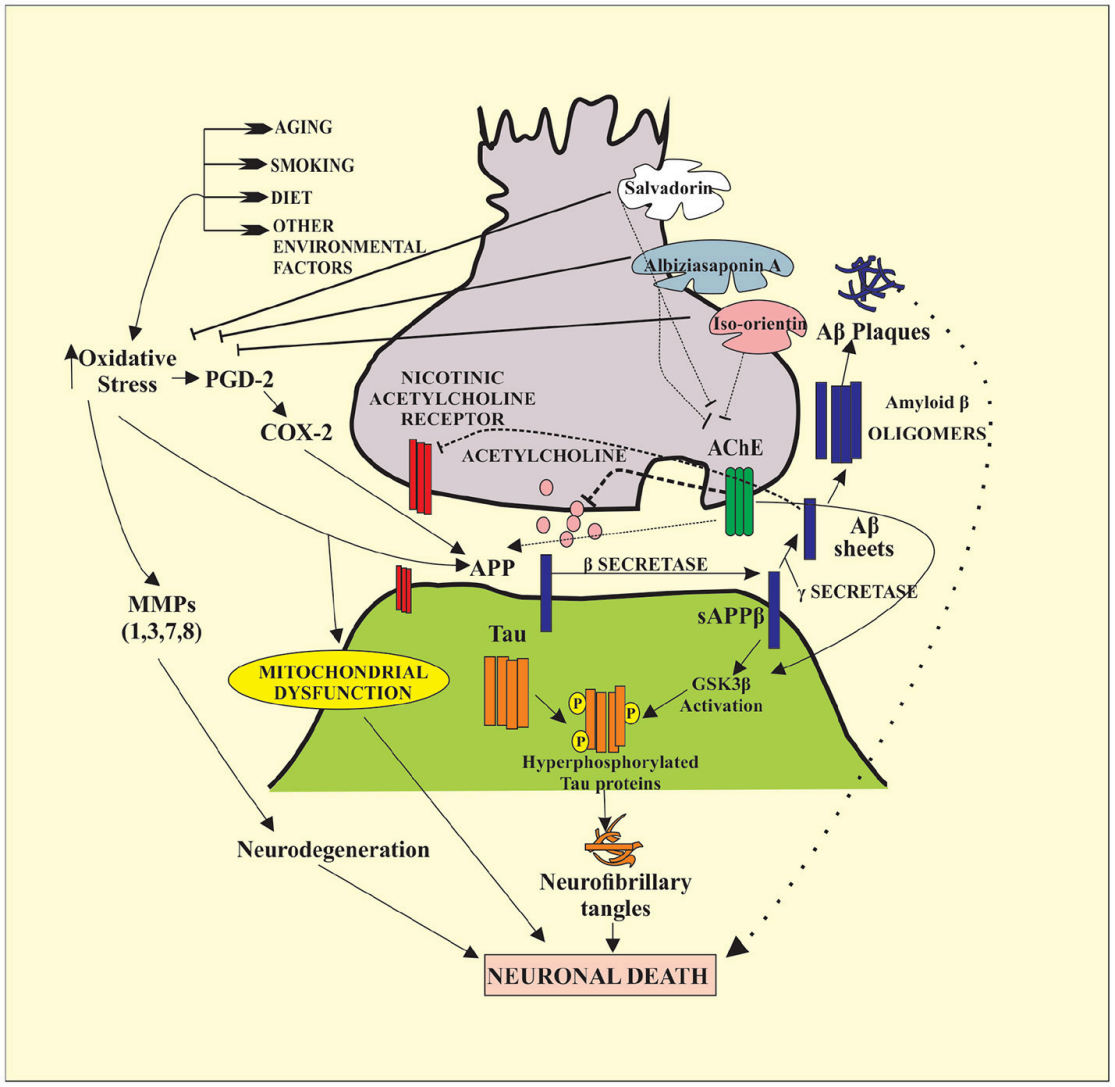
The mechanism of Alzheimer's disease (AD). It shows the role of Acetylcholine Esterase (AchE) and oxidative stress in the neurodegeneration. Oxidative stress and AchE up-regulates the activity of Amyloid precursor proteins (APPs). Moreover, oxidative stress is involved in the activation of several MMPs and enzymes cyclooxygenase-2 (COX-2). MMPs are directly responsible for the degradation of extracellular membrane (ECM) that leads to neurodegeneration. Under the action of enzyme β-secretase APPs gets converted into serum APPβ that later with the action of γ-secretase is converted into amyloid-β sheets. These amyloid-β sheets ultimately form amyloid β plaques. Alzheimer disease is often characterized with the presence of amyloid β plaques, neurofibrillary tangles, and hyperphosphorylated tau proteins. Tau proteins are hyperphosphorylated under the action of GSK3β which is activated by the activity of sAPPβ. Cumulatively, all of the discussed factors are involved in the neurodegeneration, which leads to the Alzheimer disease. Most of the drugs used in the following case are AchE inhibitors. They halt the AchE so, there will be enough neurotransmission present for the proper neuronal functioning. Likewise, in the current study, salvadorin, albiziasaponin and iso-orientin, significantly blocked the activity of AchE to cause neuroprotection.

In the present study, Albiziasaponin-A, Iso-Orientin, and Salvadorin inhibit the activity of AChE in rats with experimentally induced AD. Furthermore, the inflammatory markers and oxidative stress levels were attenuated by these three compounds in the experimental rat model of AD. The serum levels of AChE, 4-HNE, 8-OHdG, TNF-α, Iso-P2α, MDA, COX-2, and MMP-8 were significantly reduced in the groups of rats treated with these compounds. Recent studies have further emphasized the importance of inhibiting the activity of AChE and BChE enzymes in AD patients (Ayaz et al., 2015, 2017a,b). After the screening of compounds by all possible dry and wet lab techniques it explains cognitive decline as necessary complication for the emergence of AD. It also tends to explain that increasing the cholinergic tone may help in reverting cognitive dysfunction either by the help of ACh precursors or by antagonizing nicotinic receptors as shown in Figures 2–4.

**Figure 5:**
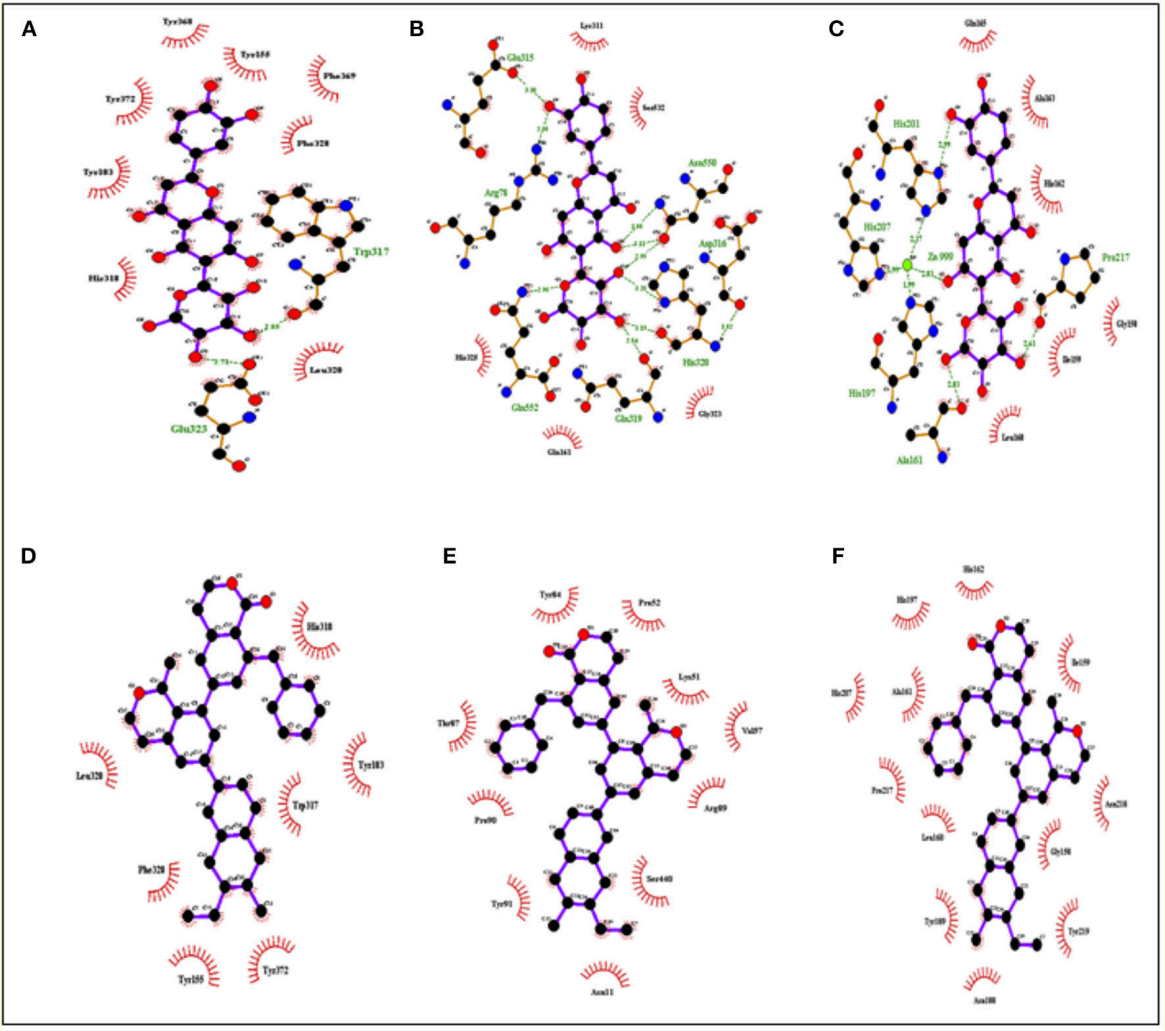
Interactions of top ranked compounds against 3 targeted proteins **(A,B)** Albiziasaponin-A, **(C,D)** Iso-orientin, and **(E,F)** Salvadorin. Ligplot showed that Atoms of compounds and the interacting residues in the standard element colors respectively Iso-orientin and Salvadorin against AChE, Cox-2 and MMP8. The Ligplot did not show any Pi-Pi interactions of the selected compounds with the respective target proteins.

Here, we have further observed a strong and significant positive correlation among different variables, AChE vs. MMP-8 (*r* = 0.823^**^), TNF-α vs. MMP-8 (*r* = 0.865^**^), 8-OHdG vs. MDA (*r* = 0.719^**^), and 4-HNE vs. MDA (*r* = 0.774^**^) in rats experimentally induced with colchicine and administered with Albiziasaponin-A, Iso-Orientin and Salvadorin. Such correlations depict that if one of the variables is increased; it might cause the increase of other positively associated factors. As described, AChE is one of the primary enzymes responsible for the neurological dysfunctions therefore, depending upon the discussed correlations it may be stated as increased inflammatory status, oxidative stress, and DNA damage may potently increase the levels of AChEs. Albiziasaponin A, Iso-orientin, and Salvadorin have caused significant reduction in both inflammatory and oxidative levels by the upregulation of antioxidant enzymes and the inhibition of AChE. More notably, Iso-orientin is a polyphenolic compound contains ortho-dihydroxyl substituent over its aromatic ring (Brown et al., 1998). It works as an antioxidant by donating its hydrogen atom to free radicals present in the cells. The role of iso-orientin in the activation of several singling cascades such as PI3K, PKC, Nrf2 pathway, and MAPK is critical for its anti-oxidant properties. For example, PI3K activates the NQO1, which leads to the release of Nrf2 from Keap1 through Nrf2-ARE cascade and subsequently increase the levels of antioxidant enzymes (Li et al., 2006) leading to neuroprotection. Such neuroprotective activities serve as an important treatment strategy for AD. Four out of five different therapies available for AD are primarily based on the inhibition of AChE. Activities of in rats experimentally induced with colchicine and administered with Albiziasaponin-A, Iso-Orientin and Salvadorin were also compared with the activity of FDA approved drugs such as donepezil, galantamine, and rivastigmine. Studies reported that galantamine binds to the nAChR that is a nicotinic receptor at the binding site, which is an additional binding site of its natural agonist ACh. This binding causes the allosteric modulation of nicotinic receptor because of the co-binding of ACh and galantamine. An *in vivo* study demonstrated that donepezil, physostigmine, and tacrine also modulate the nicotinic ACh receptor allosterically. Hence, in the present study, the molecular docking and *in vivo* studies have uncovered the anti-AD properties of Albiziasaponin-A, Iso-Orientin and Salvadorin. These phytocompounds could be used to develop synthetic medicines such as rivastigmine (Howes and Houghton, 2012; Forbes-Hernandez et al., 2016) for the treatment of AD.

## Conclusion

In the present study, both *in silico and in vivo* findings suggest potent neuroprotective roles of Albiziasaponin-A, Iso-orientin, and Salvadorin. The administration of these compounds in rats with experimentally induced AD result in the attenuation of AChE, oxidative stress, and inflammatory markers that play a significant role in the progression of AD. These results signify the potential of these phytocompounds as drugs against the progression of neurological disorders like AD. Further *in silico* and *in vivo* characterisation and validation of Albiziasaponin-A, Iso-orientin, and Salvadorin, against other important proteins implicated in the pathogenesis of AD may be essential to decipher novel mechanistic insights before taking these phytocompounds for preclinical studies.

## Ethics statement

This study was carried out in accordance with the recommendations of the University of Lahore Animal Ethics Committee. The protocol was approved by the University's Ethics Committee.

## Author contributions

AM, SW, QT-A, TJ, RR, AK, MG, SS, MR, and MN designed the experiments. AM, SW, QT-A, TJ, RR, AK, MG, and SS conducted the experiments. MR, MN, PP, KG, and MA-Q analyzed the data. MR, MN, PP, AM, SW, QT-A, TJ, RR, AK, MG, SS, KG, and MA-Q wrote the paper. MR, MN, and PP proposed the research idea. All authors contributed to the editing of the paper and the scientific discussions.

### Conflict of interest statement

The authors declare that the research was conducted in the absence of any commercial or financial relationships that could be construed as a potential conflict of interest. The reviewer NK and handling Editor declared their shared affiliation.
